# Supplementary material for: Liver Function in Patients with Long-Term Coronavirus Disease 2019 of up to 20 Months: A Cross-Sectional Study
Source: Int J Environ Res Public Health. 2023 Mar 28;20(7):5281. doi: 10.3390/ijerph20075281 (PMC10094195; doi:10.3390/ijerph20075281)
Supplement: Supplementary file 1 [file ijerph-20-05281-s001.zip › ijerph-2244587-supplementary.pdf]

Supplementary Table S1. Adopted reference values for clinical and laboratory exams.

Exam	Reference parameter (maximum and minimum value)
ALT (U/L)	22-29
AST (U/L)	18-25
LDH (U/L)	230-460
ALP (μg/L)	40-190
GGT (μg/L)	7-32[a] | 11-50[b]
PT (seconds)	11-15
Ferritin (ng/mL)	20-300[a] | 30-300[b]
CRP	Positive | negative
ESR (mm)	0-20[a] | 0-30[b]
TB (mg/dL)	0-1
DB (mg/dL)	0-0,3
IB (mg/dL)	0-0,8
Albumin (g/dL)	3,5-4,8

[a]Women. [b]Male. ALT: Alanine aminotransferase. AST: Aspartate aminotransferase. LDH: Lactic dehydrogenase. TB: Total bilirubin. DB: Direct bilirubin. IB: Indirect bilirubin. ALP: Alkaline phosphatase. GGT: gamma-glutamyl transferase. CRP, C-reactive protein. PT: Prothrombin time. ESR: Erythrocyte sedimentation rate.
